# Altered Dopamine Metabolism and Response to Treatment with Levodopa/Carbidopa in MCT8 Deficiency

**DOI:** 10.1002/mds.70093

**Published:** 2025-10-27

**Authors:** Fabio Bruschi, Ylenia Vaia, Clara E. Antonello, Marco Spada, Francesco Porta, Cristina Marinaccio, Claudia Carducci, Thomas Opladen, Jacopo Sartorelli, Federica Maria Zibordi, Daniele Ghezzi, Francesco Nicita, Davide Tonduti

**Affiliations:** ^1^ Unit of Pediatric Neurology, COALA (Center for Diagnosis and Treatment of Leukodystrophies) V. Buzzi Children's Hospital Milan Italy; ^2^ Department of Biomedical and Clinical Sciences Università degli Studi di Milano Milan Italy; ^3^ Department of Pediatrics University of Torino Torino Italy; ^4^ Department of Clinical and Biological Sciences University of Torino Torino Italy; ^5^ Child and Adolescence Neuropsychiatry Service, Department of Child Pathology and Cure Regina Margherita Children's Hospital Turin Italy; ^6^ Department of Experimental Medicine Sapienza University of Rome Rome Italy; ^7^ Heidelberg University Medical Faculty, Heidelberg Center for Pediatric and Adolescent Medicine Department I Division of Pediatric Neurology and Metabolic Medicine Heidelberg Germany; ^8^ Unit of Muscular and Neurodegenerative Diseases Bambino Gesù Children's Hospital, IRCCS Rome Italy; ^9^ Department of Pediatric Neuroscience Fondazione IRCCS Istituto Neurologico Carlo Besta Milan Italy; ^10^ Unit of Medical Genetics and Neurogenetics Fondazione IRCCS Istituto Neurologico Carlo Besta Milan Italy; ^11^ Department of Pathophysiology and Transplantation University of Milan Milan Italy

**Keywords:** hypothyroidism, Allan‐Herndon‐Dudley syndrome, SLC16A2, MCT8, parkinsonism, dystonia, neurotransmitter

## Abstract

**Background:**

Allan‐Herndon‐Dudley syndrome (AHDS)/monocarboxylate transporter 8 (MCT8) deficiency is a rare X‐linked encephalopathy caused by *SLC16A2* variants, impairing thyroid hormone (TH) transport into the brain. This leads to early central nervous system (CNS) TH deficiency, affecting brain maturation. Dopaminergic circuit involvement is suggested by both pathophysiology and clinical features, reminiscent of infantile parkinsonism.

**Objective:**

This study investigates dopamine metabolism and levodopa/carbidopa response in MCT8 patients.

**Methods:**

We retrospectively and prospectively collected clinical, genetic, and neuroimaging data, performed cerebrospinal fluid (CSF) biogenic amine analyses, and conducted neurological assessments before and after the levodopa trial (10 mg/kg/day).

**Results:**

Ten patients exhibited developmental delay, spasticity, and infantile parkinsonism. CSF analysis showed reduced homovanillic acid in 3/10 patients, with 7/10 in the lowest quartile. Levodopa improved parkinsonism and reactivity in 7/10 patients.

**Conclusions:**

Our findings confirm dopaminergic involvement in AHDS and show that levodopa/carbidopa effectively treats extrapyramidal symptoms. Further investigations could differentiate presynaptic and postsynaptic defects to optimize dopaminergic therapy. © 2025 The Author(s). *Movement Disorders* published by Wiley Periodicals LLC on behalf of International Parkinson and Movement Disorder Society.

Allan‐Herndon‐Dudley syndrome (AHDS), or monocarboxylate transporter 8 (MCT8) deficiency, is a rare X‐linked encephalopathy affecting approximately 1 in 70,000 males. It is caused by pathogenic variants in *SLC16A2*, which codes for the MCT8, a crucial membrane transporter for thyroid hormones (THs).^1^, ^2^ AHDS presents with severe developmental delay/intellectual disability, axial hypotonia, combined pyramidal and extrapyramidal signs, and often epilepsy. In AHDS, the peripheral plasma profile is characterized by elevated T3, reduced T4, and often normal thyroid‐stimulating hormone, leading to peripheral thyrotoxicosis, which can result in significant cardiac and metabolic complications.^1^, ^3^

Patients exhibit markedly low central nervous system (CNS) TH levels and CNS hypothyroidism, which is thought to underline the severe neurological manifestations seen in patients with AHDS.^2^, ^4^, ^5^ Among these, movement disorders (MDs) have been frequently reported. Recently, we investigated MDs in a large cohort of patients with AHDS and found that hypokinesia was the most prevalent, affecting more than two‐thirds of subjects and having a major impact on patients' global disability.^6^ In line with the classification of childhood‐onset parkinsonism proposed by Leuzzi et al,^7^ these patients could be clinically diagnosed with developmental parkinsonism even if evidence for dopaminergic circuitry impairment or dopamine depletion in MCT8 deficiency is currently lacking.

Clinical trials of TH analogues have not shown a significant impact on the neurological manifestations of the disease, including MDs.^8^

Wilpert et al^9^ investigated dopamine metabolism in patients with AHDS, found cerebrospinal fluid (CSF) biogenic amines abnormalities suggestive of an isolated pathway impairment, and observed a favorable response to levodopa (l‐dopa)/carbidopa. With our study we also sought to investigate dopamine metabolism in patients with AHDS and further evaluate the efficacy of l‐dopa in managing extrapyramidal signs.

## Methods

We recruited patients with a genetically confirmed diagnosis of AHDS followed at three different tertiary centers in Italy with expertise in pediatric neurology: COALA (Center for Diagnosis and Treatment of Leukodystrophies) at Vittore Buzzi Children's Hospital in Milan, IRCCS Istituto Neurologico Fondazione Carlo Besta in Milan, and Bambino Gesù Children's Hospital in Rome.

Demographic, clinical, genetic, and neuroimaging data were systematically collected in an electronic database. The study adhered to the Declaration of Helsinki and received Institutional Review Board approval, with written informed consent obtained from parents or legal representatives.

Patients underwent comprehensive assessment for MDs using clinical evaluation, standardized scales, and video recordings, before and after drug titration and then every 3 months. For patients who were evaluated restrospectively, we relied on data in clinical charts.

All patients had lumbar punctures for CSF biogenic amine metabolite analysis (Table 2).

l‐Dopa was administered, starting at 1 mg/kg/day, and slowly titrated up to a target posology of 10 mg/kg/day. Treatment response was evaluated clinically, through parent reports, and qualitatively scored using the Clinical Global Impression–Improvement scale (CGI‐I).

## Results

### Clinical Data

We recruited, both retrospectively (3 patients) and prospectively (7 patients), 10 male patients with AHDS. Clinical and genetic data are summarized in Supporting Information Table S1.

At baseline, all patients exhibited hypokinetic MDs, including hypobradykinesia, hypomimia, and low motor initiative. Nine patients presented a severe phenotype characterized by absent postural control, global hypokinesia, mild to moderate dystonia, pyramidal signs, axial hypotonia, and low social interaction while alert. One patient presented a milder phenotype as he achieved autonomous walking (standing up at 2 years, autonomous walking at 3.5 years); his clinical picture was reminiscent of a classic parkinsonian syndrome except for the absence of resting tremor, which was associated with spasticity. Seven patients presented with startle reaction and 2/10 with paroxysmal phenomena. Motor and functional scales scores are reported in Table 1.

**TABLE 1:** Neuromotor and functional assessment before and after levodopa/carbidopa titration (10 mg/kg/day; 3‐ and 6‐ to 9‐month follow‐up)

	Patient 1 (13.3 y)			Patient 2 (5.9 y)			Patient 3 (14 y)			Patient 4 (6.1 y)		Patient 5 (5.5 y)			Patient 6 (18 y)		Patient 7 (1.5 y)		Patient 8 (1 y)		Patient 9 (0.75 y)		Patient 10 (1 y)	
Scales	Basal	3 mo post‐LD	6 mo post‐LD	Basal	3 mo post‐LD	9 mo post‐LD	Basal	3 mo post‐LD	6 mo post‐LD	Basal	3 mo post‐LD	Basal	3 mo post‐LD	6 mo post‐LD	Basal	3 mo post‐LD	Basal	3 mo post‐LD	Basal	3 mo post‐LD	Basal	3 mo post‐LD	Basal	3 mo post‐LD
GMFM‐88 (%)																								
Lying and rolling (A)	9.8	17.6	19.6	17.6	27.5	27.5	3.9	17.6	17.6	17.5	27.5	2	2	2	100	100	31.37	25.49	33.33	27.45	9.8	NA	na	na
Sitting (B)	8.3	8.3	8.3	5	6.7	6.7	10	11.7	11.7	5	5	0	0	0	100	100	1.6	1.6	0	0	0	NA	na	na
Crawling and kneeling (C)	0	0	0	0	0	0	0	0	0	0	0	0	0	0	92.9	100	0	0	0	0	0	NA	na	na
Standing (D)	0	0	0	0	0	0	0	0	0	0	0	0	0	0	76.9	76.9	0	0	0	0	0	NA	na	na
Walking, running, jumping (E)	0	0	0	0	0	0	0	0	0	0	0	0	0	0	52.8	56.9	0	0	0	0	0	NA	na	na
**Total score**	3.6	5.2	5.6	4.5	6.8	6.8	2.8	5.9	5.9	4.5	6.5	0.4	0.4	0.4	84.5	86.8	6.59	5.5	6.66	5.49	1.96	NA	na	na
EDACS	4	4	4	4	4	4	5	5	5	3	3	5	5	5	1	1	3	3	3	3	3	NA	5	5
CFCS	5	5	4	5	5	4	5	5	4	5	5	5	5	5	3	3	4	4	5	5	4	NA	5	5
MACS	5	5	5	5	5	5	5	5	5	5	5	5	5	5	3	3	5	5	5	5	5	NA	5	5
GMFCS	5	5	5	5	5	5	5	5	5	5	5	5	5	5	2	2	5	5	4	4	5	NA	5	5
GMFCS‐MLD	5	5	5	5	5	5	5	5	5	6	6	6	6	6	1	1	6	6	5	5	6	NA	5	5
BADS	12	12	10	16	16	16	na	na	na	14	na	16	16	23	5	na	17	13	15	15	6	NA	na	na
BFMDRS																								
Dystonia movement scale	39	39	35	25.5	37.5	36	15.5	23.5	23.5	21	22.5	63	63	84	2	2	50.5	38.5	na	na	na	NA	na	Na
UPDRS part III	NA	NA	NA	NA	NA	NA	NA	NA	NA	NA	NA	NA	NA	NA	48	47	NA	NA	NA	NA	NA	NA	NA	NA

*Note*: A detailed neuromotor and functional assessment was performed using standardized scales.

Abbreviations: GMFM‐88, Gross Motor Function Measure; NA, not applicable; na, not available; EDACS, Eating and Drinking Ability Classification System; CFCS, Communication Function Classification System; MACS, Manual Ability Classification System; GMFCS, Gross Motor Function Classification System; GMFCS‐MLD, Gross Motor Function Classification System–Metachromatic Leukodystrophy; BAD, Barry‐Albright Dystonia Scale; BFMDRS, Burke‐Fahn‐Marsden Dystonia Rating Scale; UPDRS‐III, Unified Parkinson's Disease Rating Scale part III.

### CSF Profiles

CSF collection was performed in all patients at a mean age of 6.9 (0.7–17) years. Biogenic amines were measured, and results are summarized in Table 2. CSF homovanillic acid (HVA), a dopamine metabolite, was pathologically low in three patients; in 7/10, levels were in the lowest quartile of the age‐specific reference range. One patient (patient 8) with initially altered HVA normalized after l‐dopa/carbidopa. 5‐Hydroxyindoleacetic acid (5‐HIAA) was normal in all but one patient (elevated), with no reported concomitant serotoninergic drug use. The HVA/5‐HIAA ratio was normal across all patients.

**TABLE 2:** CSF biogenic amine neurotransmitter analysis

ID	Age at CSF analysis (y)	HVA	Reference range	5HIAA	Reference range	HVA/5HIAA	Reference range
Patient 1	13	117^a^	190–507^a^	77	75–203	1.5	1.5–3.9
Patient 2	5.3	407	313–824	149	130–362	2.7	1.5–4.1
Patient 3	13.5	136^a^	190–507^a^	70	75–203	1.9	1.5–3.9
Patient 4	5	346	313–824	172	130–362	2.01	1.5–3.4
Patient 5	5.1	142	88–178	208	144–801	1.5	1.5–3.5
Patient 6	17	286	98–450	172^a^	45–135^a^	1.66	1.5–3.5
Patient 7	1.5	292	236–867	255	97–367	1.14	1.5–3.5
Patient 8	1	234^a^	364–870^a^	155	155–319	1.5^a^	1.6–3.9^a^
Patient 8 after trial with levodopa/carbidopa	2	461	364–870	214	155–359	2.2	1.6–3.9
Patient 9	0.75	492	302–845	293	152–462	1.63	1.5–3.5
Patient 10	1	501	295–932	313	114–336	1.6	1.5–3.5

^a^ Pathological levels of HVA (patient 1, patient 3) and 5HIAA (patient 6) (all values are nmol/l).

*Abbreviations*: CSF, cerebrospinal fluid; HVA, homovanillic acid; 5HIAA, 5‐hydroxyindolacetic acid.

### Levodopa/Carbidopa Trial

l‐Dopa/carbidopa was slowly titrated up to 10/2.5 mg/kg/day over 3 months in nine patients; one patient died at the age of 15.5 years, so we collected data on only biogenic amines and could not evalute response to treatment. Therapeutic benefit was observed only with a dosage exceeding 7 to 8 mg/kg/day. At this dosage, seven of the nine patients experienced better motor and nonmotor symptoms, including enhanced attention and a greater response to environmental stimuli, as indicated by better eye contact and social interaction, and reduced hypomimia (Video S1). In all nine cases, parents referred the same impression reported by secondary caregivers (physical therapists, speech therapists, teachers), who also described an improvement of hypomimia. l‐Dopa treatment led also to decreased hypokinesia/bradykinesia in eight of nine patients (Video S2). GMFM‐88 (Gross Motor Function Measure) improved in five of eight patients, showing a mean 50.69% increase in total score, primarily in Lying/Rolling (A) and Sitting subscales (B). Two patients had slight GMFM‐88 declines, and one showed no benefit. Despite GMFM‐88 score increases, no congruent improvements were observed across functional scales (Table 1). As expected, the increased motor activity corresponded to increased evidence of action dystonia, reflected by an 11.7% mean increase in BFMDRS scores, even if CGI‐I showed four patients as “much improved,” two as “minimally improved,” and three as “no change.”

**VIDEO 1:** Hypomimia and reactivity to environmental stimuli improvement after levodopa/carbidopa introduction at 3‐month follow‐up (10 mg/kg/day).

**VIDEO 2:** Hypobradkinesia and motor initiative improvement after levodopa/carbidopa introduction at 3‐month follow‐up (10 mg/kg/day). As expected, dystonic movements and posturing increase after drug titration; as per definition, dystonia is also induced by movement.

For three patients, a 6‐month follow‐up after l‐dopa introduction was available. Of these, one patient showed a further increase in GMFM‐88 scores; one patient, who had already shown no response at 3 months, did not exhibit any score increase.

In addition, for one patient, a 9‐month follow‐up was available, confirming sustained responsiveness to l‐dopa; GMFM‐88 score remained unchanged compared with the 3‐month evaluation, and clinical benefits persisted.

## Discussion

We describe in this report a series of 10 patients diagnosed with AHDS, for which MDs were a common finding at neurological evaluation. All patients presented a clinical picture suggestive of developmental parkinsonism. In line with our previous results, parkinonian features were generally far more pronounced than dystonia and seemed to contribute significantly to the overall functional disability, alongside pyramidal signs and severe cognitive impairment.^6^

To better investigate the pathophysiology of MDs in these patients, we conducted a CSF biogenic amine analysis showing HVA below or at lower limits of the reference range in almost all subjects, thus strengthening recent observations by Wilpert et al.^9^ These concentrations were significantly higher than those typically observed in primary neurotransmitter disorders, yet consistent with a secondary defect.

In our cohort, the trial with l‐dopa/carbidopa demonstrated a positive response in seven patients, particularly in terms of increased responsiveness to environmental stimuli and interaction. All patients exhibited an improvement in hypomimia, with more evident facial expressions such as smiles in response to tactile or verbal stimuli from parents or physicians. Motor improvements included enhanced spontaneous activity and greater initiative in most patients, even with persistent spasticity, dystonia, and severe axial hypotonia. Notably, increased spontaneous motor activity was accompanied by slightly more prominent dystonic components, which was an expected finding because dystonia can worsen with voluntary movements.

Interestingly, in our cohort, l‐dopa–responsive patients seemed to maintain benefit from the drug both at 6 and 9 months after introduction. In our cohort, we did not observe any side effect.

THs play a pivotal role in human brain development and functionality. In fact, a lack of THs during embryonic, fetal, and immediate postnatal stages leads to significant alterations in CNS development, affecting both CNS structure and function.^10^ Thyroid organogenesis starts by the seventh week of gestation, during which the MCT8 transporter, crucial for TH action at the CNS level, is fully expressed in the cerebral cortex, with mRNA levels comparable with those of adulthood.^11^, ^12^ Immunohistochemistry studies in murine models showed that MCT8 is widely expressed in the CNS, highlighting the essential role of THs in developing neural structures and circuits during both intrauterine and early postnatal life. In particular, THs have been shown to be key factors in promoting dopaminergic differentiation of primary murine neural stem cells and rat neural progenitor cells, as well as neural progenitors derived from human embryonic stem cells in vitro, specifically in relation to the basal ganglia.^13^

TH and MCT8 are essential not only for proper fetal development of extrapyramidal circuits but also for their correct postnatal function; they appear to be involved in dopamine synthesis and trafficking. In fact, in neonatal hypothyroidism, reduced activity of tyrosine hydroxylase, an enzyme responsible for dopamine synthesis and turnover, has been observed^10^, ^14^; in addition, alterations in axonal transport of dopamine vesicles from the substantia nigra to the striatum have been demonstrated because of altered expression levels of various tubulin isoforms.^10^, ^15^ Furthermore, the expression of MCT8 in both D1‐ and D2‐medium spiny neurons of the basal ganglia suggests that its impaired function may disrupt the precise balance of the striatal microcircuitry, which includes the direct (D1) and indirect (D2) pathways, thus contributing to the pathophysiology of MDs.^16^

In summary, the pathophysiology of the extrapyramidal signs characteristic of AHDS is complex, multifactorial, and not yet fully understood. Basal ganglia dysfunction may arise from an early disruption of the normal developmental process of these structures during the gestational period, abnormal dopamine synthesis and trafficking, thus suggesting considering AHDS a fetopathy.

Basal ganglia involvement reflects in MDs typical of this syndrome, particularly parkinsonism and dystonia, for which therapy with l‐dopa should be considered.

This study was limited by several factors, including its small sample size because of disease rarity. The evaluation of environmental reactivity and attention was also challenged by a reliance on subjective measures and the absence of standardized scales for childhood parkinsonism. Furthermore, the partly retrospective nature of the study led to incomplete data for some patients. Future research should expand the sample size, develop specific standardized scales, and conduct in vivo studies of the dopaminergic system using nuclear medicine techniques to understand pathophysiology and guide new treatments.

## Author Roles

(A) Study conception and design, (B) study execution, (C) data analysis, (D) writing of the first draft, (E) review and critique, (F) editing of final version of the manuscript.

F.B.: A, B, C, D, E, F.

D.T.: A, B, C, D, E, F.

Y.V.: B, E.

C.E.A.: B, E.

M.S.: B, E.

F.P.: B, E.

C.M.: B, E.

C.C.: B, E.

T.O.: B, E.

J.S.: B, E.

F.M.Z.: B, E.

D.G.: B, E.

F.N.: B, E.

## Financial Disclosures for the Previous 12 Months

D.T. participated in scientific advisory with Medscape, Icometrix, Inizio Evoke, and Regulatory Pharma Net.

## Supporting information


**Table S1.** Clinical, genetic and instrumental data of the MCT8 cohort.

## Data Availability

Upon request, the corresponding author can provide access to the data supporting this study's findings.
